# Temporal Changes of Microarchitectural and Mechanical Parameters of Cancellous Bone in the Osteoporotic Rabbit

**DOI:** 10.1155/2015/263434

**Published:** 2015-03-31

**Authors:** Xin-Xin Wen, Chao Xu, Fa-Qi Wang, Ya-Fei Feng, Xiong Zhao, Ya-Bo Yan, Wei Lei

**Affiliations:** Department of Orthopedics, Xijing Hospital, Fourth Military Medical University, Changle West Road No. 15, Xi'an, Shaanxi 710032, China

## Abstract

This study was aimed at elucidating the temporal changes of microarchitectural and mechanical parameters of cancellous bone in the osteoporotic rabbit model induced by ovariectomy (OVX) combined with glucocorticoid (GC) administration. Osteoporotic (OP) group received bilateral OVX combined with injections of GC, while sham group only received sham operation. Cancellous bone quality in vertebrae and femoral condyles in each group was assessed by DXA, *μ*CT, nanoindentation, and biomechanical tests at pre-OVX and 4, 6, and 8 weeks after injection. With regard to femoral condyles, nanoindentation test could detect significant decline in tissue modulus and hardness at 4 weeks. However, BMD and microarchitecture of femoral condylar cancellous bone changed significantly at 6 weeks. In vertebrae, BMD, microarchitecture, nanoindentation, and biomechanical tests changed significantly at 4 weeks. Our data demonstrated that temporal changes of microarchitectural and mechanical parameters of cancellous bone in the osteoporotic rabbit were significant. The temporal changes of cancellous bone in different anatomical sites might be different. The nanoindentation method could detect the changes of bone quality at an earlier stage at both femoral condyle and vertebra in the osteoporotic rabbit model than other methods (*μ*CT, BMD).

## 1. Introduction

Osteoporosis is a skeletal disease characterized by low bone mass, microarchitectural deterioration of bone tissue leading to increased bone fragility, and a consequent increase in the risk of fracture [1]. Animal models could provide comprehensive information about the etiology of osteoporosis and facilitate the development of new treatments for osteoporosis [2]. In the preclinical testing of agents intended to prevent or treat osteoporosis, US FDA recommends that agents should be evaluated at least in two animal species [3].

One such suitable experimental animal is rabbit. Since rabbits achieve skeletal maturity at approximately 6 months of age, show significant intracortical remodeling [4, 5], and have faster bone turnover than other rodents, even than primates, a significant bone loss can be induced within a short period of time [4–7], and their size renders the insertion of conventional implants easily [8]. These aspects turn this animal into a promising model to study osteoporosis and to investigate and test anabolic drugs. In spite of these potential advantages, experimental models of osteoporosis in rabbits have not been fully characterized.

Currently, the osteoporotic rabbits were established to study osteoporosis only in a few studies. In trying to reproduce postmenopausal osteoporosis, estrogenic deprivation has been the most commonly used experimental method for osteoporosis in animals. Jensen et al. [9] utilized OVX rabbits in their study; the duration of estrogen withdrawal was 27 weeks. With regard to vertebral cancellous bone, OVX significantly reduced bone volume and trabecular numbers, significantly increased trabecular space, and did not affect trabecular thickness. In another study, there was a significant reduction of lumbar vertebral BMD in OVX rabbits compared to the sham group at 13 weeks after surgery [10]. With regard to the cortical bone, the femur was both larger and weaker 16 weeks after surgery in the OVX rabbits [11]. But the time length to establish this osteoporotic rabbit model was quite long.

Castañeda et al. [6, 7] proposed an experimental model of osteoporosis in rabbits induced by a combination of OVX and GC administration. This combination method could establish an osteoporotic model in a quite short time length. Li et al. [12] established the rabbit model of osteoporosis by a combination of OVX and GC administration and to explore the evidence of regular alteration of bone quality in osteoporosis, MR spectroscopy, *μ*CT, and histopathology were used. Baofeng et al. [13] and Liu et al. [14] assessed the microarchitecture and biomechanical strength of cancellous and cortical bone in OVX and GC treated rabbits. Osteoporotic model in rabbits was built up successfully 4 weeks after OVX surgery. To a certain extent, the combination of OVX and GC was expected to mimic the circumstances seen in postmenopausal women receiving GC therapy [15]. However, few studies focused on the temporal changes of bone quality, which might provide us with more information about the development of osteoporosis in this rabbit model.

Recently, the technique of nanoindentation has been employed to determine the nanomechanical properties of bone tissue. Nanoindentation examines bone at the material level and can probe the biomechanical properties at the microscale [16]. To our knowledge, the nanomechanical alteration in cancellous bone of the osteoporotic rabbit induced by OVX + GC (OVX combined with GC administration) was not determined earlier.

Monitoring bone quality over time in osteoporotic animal model may provide new insight into bone remodeling in osteoporosis diagnosis and treatment [17, 18]. Therefore, the purpose of this study was to explore the temporal changes of cancellous bone quality in an osteoporotic rabbit. For this purpose, we aimed to detect the temporal changes on cancellous bone in terms of BMD, microarchitecture, nanomechanical, and biomechanical properties during the development of osteoporosis in rabbits.

## 2. Methods

### 2.1. Animals

40 female, 7-month-old (3.16 ± 0.27 kg), skeletally mature, New Zealand white rabbits were included in the present study (Fourth Military Medical University, Xi'an, Shaanxi, China). These experiments were approved by the local ethics committee (Fourth Military Medical University Ethics Committee) and the guidelines for care and use of animals were followed. The rabbits were kept individually in cages and maintained on a “12-hour light/12-hour dark” cycle at room temperature with ad libitum access to water and standard commercial rabbit chow.

### 2.2. Osteoporotic Rabbit Model

After the rabbits were acclimatized to the new conditions for 2 weeks, they were randomly allocated into a sham operation group (sham group, *n* = 20) and an osteoporotic model group (OP group, *n* = 20). Five rabbits in each group were euthanized to acquire baseline data (BMD, *μ*CT, nanoindentation, and biomechanical test) with no treatment before OVX surgery. Rabbits in group OP received bilateral OVX combined with daily intramuscular injections of MPS (methylprednisolone sodium succinate) (Pfizer Manufacturing Belgium NV) for 4, 6, and 8 weeks at a dose of 1.0 mg/kg/day. The daily MPS injections in the group OP were administered 2 weeks after OVX [7]. For the sham-OVX operation in the sham group, the ovaries were held up and then returned to their original positions. Antibiotics (ampicillin, 0.1 g/kg/day, China) were administered before surgery and for 5 days after surgery to prevent infection. All animals were weighed and the doses of MPS were adjusted on a weekly basis. DXA of the third and fourth lumbar vertebra (L3 and L4) was carried out at 4, 6, and 8 weeks after injection in both groups (five rabbits at each time point in each group). After accomplishing the DXA examination, the rabbits were euthanized by intravenous administration of an overdose of Sumianxin II (Changchun Veterinary Institute of Military Medical Academy of Sciences, China) at each time point (MPS injection for 4 weeks, 6 weeks, and 8 weeks) in each group. After death, the lumbar spine (from the first lumbar vertebrae to the fifth lumbar vertebrae: L1–L5) and femora (left and right) were dissected, soaked in 0.9% saline solution gauze, and frozen at −80°C in plastic bags for the further examinations.

### 2.3. DXA

**Figure 1:**
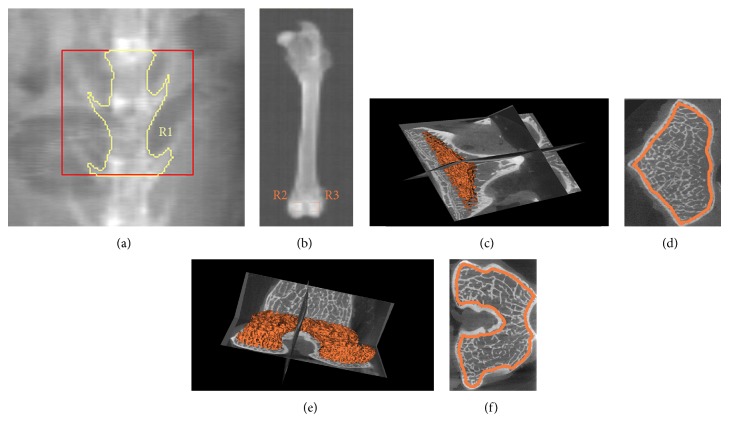
DXA and *μ*CT measurement of vertebrae and femoral condyles. (a) DXA measurement of vertebrae. R1: ROI of L3 and L4. (b) DXA measurement of femoral condyles. R2 and R3: ROI of femoral condyles (7 mm × 7 mm ROI). (c) The 3D reconstruction of the ROI in vertebrae in *μ*CT measurement. (d) The ROI on the axial section of vertebrae in *μ*CT measurement. (e) The 3D reconstruction of the ROI in femoral condyles in *μ*CT measurement. (f) The ROI on the axial section of femoral condyles in *μ*CT measurement. DXA: dual-energy X-ray absorptiometry; ROI: regions of interest.

DXA analysis was performed with a Hologic Discovery Wi using linear fan beam technology (analysis software version 12.7; Hologic Inc., Bedford, MA, USA); switching between two X-ray potentials (100 and 140 kVp) from an X-ray source mounted beneath the subject was done and the small animal-scanning mode was applied for all scanning. Before the rabbits were euthanized at each time point (pre-OVX and MPS injection for 4 weeks, 6 weeks, and 8 weeks) in each group, the BMD of lumbar vertebrae was determined. Measurements were carried out in vivo with the animals placed in the supine decubitus position under general anesthesia. A point, 3 cm below the navel, was considered to be the external guide to focus the DXA beam at about L3-L4 [6]. Mean absorptiometry values of L3 and L4 were calculated to represent the BMD of lumbar vertebrae (Figure 1(a)). After the rabbits were euthanized, the left femora underwent BMD scans in vitro. In each femoral condyle, two cancellous bone-rich scanning regions of interest (ROI) were positioned (7 mm × 7 mm, Figure 1(b)) to represent the BMD of femoral condyles.

### 2.4. *μ*CT Analysis

The harvested bones were frozen at −80°C until the time of scanning by *μ*CT (Healthcare Explore Locus, GE Medical Systems, Milwaukee, USA). The specimens were thawed at room temperature before *μ*CT scan and kept moist by saline during all scanning procedures. L5 vertebrae were obtained and removed from the appendixes. Right femoral condyles were harvested with the diaphysis cutoff. The images were obtained using the following parameters: (a) 80 kV as the X-ray tube voltage; (b) 80 *μ*A as the anode current; (c) 3000 ms as the shutter speed; (d) 2 as the binning factor; and (e) 0.5° as angle of increment. The *μ*CT system was used at a spatial resolution of 14 *μ*m, and CT images were reconstructed in 1024 × 1024-pixel matrices. Microview v2.1.2 software was used. Cancellous bones in the vertebral body and femoral condyles were evaluated. The ROI of L5 vertebrae was defined as follows: (1) on the axial section, a region whose contour was drawn directly adjacent to the endocortical boundary was established; (2) the bottom of the ROI was adjacent to the endplate in the caudal end of vertebral body; and (3) the height of the ROI was 2 mm. The ROI of femoral condyles was defined as follows: (1) in the axial section, a region whose contour was drawn directly adjacent to the endocortical boundary was established; (2) the bottom of the ROI was adjacent to the intercondylar fossa; and (3) the height of the ROI was 2 mm. The position of the ROI in vertebrae and femoral condyles was shown in Figure 1. Bone volume/total volume (BV/TV), trabecular number (Tb.N), trabecular thickness (Tb.Th), trabecular separation (Tb.Sp), structure model index (SMI), and bone surface/bone volume (BS/BV) were determined [19].

### 2.5. Nanoindentation

L4 vertebrae of the all the rabbits under investigation were used for the nanoindentation. The right femoral condyles, scanned by *μ*CT, were also used for nanoindentation. The bone tissues were kept frozen at −80°C until the beginning of the specimen preparation and between the procedure steps. The vertebral bodies and femoral condyles were cut transversally, followed by removal of the bone marrow using a water jet (Teledyne Water Pit, Fort Collins, CO, USA) and an ultrasonic bath. All the specimens were then dehydrated in a series of alcohol (70%, 80%, 95%, and 100%) to avoid problems which can be caused by surface liquid films in identifying the point of first contact during indenter approach to the specimen surface. After dehydration, the specimens were embedded in epoxy resin to provide support for the porous network and then metallographically polished to produce the smooth surfaces needed for nanoindentation testing, first with silicon carbide abrasive papers (Starcke GmbH & Co. KG, Melle, Germany) of decreasing grit size (600, 800, and 1200 grit) and finally with diamond suspensions (0.3 and 0.05 *μ*m particle size) embedded in soft polishing cloths (Buehler, Lake Bluff, IL). The specimens were washed in deionized water between each polishing step to remove debris, taking care that the surface of the bone was not demineralized. The nanoindentation was performed using a TriboIndenter (HysitronTI90, Minneapolis, MN) with a standard Berkovich tip; the procedure can be described as follows: after the identification of the surface, the tip was loaded into the sample at a rate of 50 *μ*N/s, held at a maximum load of 1000 *μ*N for 10 s, and unloaded at 50 *μ*N/s. After removing 85% of the maximal load, constant load was held for 100 s to measure drifting of the displacement transducer due to both the thermal effect on the transducers capacitor and the viscous effect of the bone tissue behavior. Any indentations close to the mounting resin were removed from the data set to minimize effects of embedding on the measurements. Approximately six indentations were made in each feature. Bone tissue elastic modulus (*E*) and hardness (*H*) were calculated from the unloading segment of the load-displacement curve according to the method of Oliver and Pharr [20]. Mean values for the tissue elastic modulus and the hardness of bone tissue were calculated for each specimen.

### 2.6. Biomechanical Tests

The specimens were thawed before mechanical tests and kept moist during all handling and test procedures. Compressive test was performed on L5 vertebrae. Prior to the compressive test on vertebrae (L5), the endplates, spinous process, transverse, and articulate processes were cut with a low-speed precision saw (Isomet 1000, Buehler, Lake Bluff, Illinois) with continuous irrigation to obtain parallel surfaces. By these procedures, a central part of the vertebral body with parallel ends was obtained, consisting of a central trabecular core and a compact shell. The vertebral samples were placed centrally between two parallel steel plates attached to the materials-testing machine (MTS 858 System Inc., MN, USA) and compressed at a constant strain rate of 0.01/s until failure. For the vertebral body in compression, stress was calculated as the applied force divided by the cross-sectional area of the specimen [21]. After compressive test, the stress-strain curve was depicted. Elastic modulus and strength variables (ultimate stress and yield stress) were calculated using standard engineering formula from the stress-strain curve.

### 2.7. Statistical Analysis

All data were processed with the statistical system SigmaPlot 12.5 (Systat Software, Inc., San Jose, California, USA) and expressed as mean ± SD. Differences in BMD, microarchitectural parameters, nanomechanical parameters, and macromechanical parameters between two groups at the same time point were compared with independent-samples *t*-test. Differences of those parameters among different time points in the same group were performed with the one-way ANOVA test. *P* < 0.05 was considered statistically significant.

## 3. Results

### 3.1. BMD

**Figure 2:**
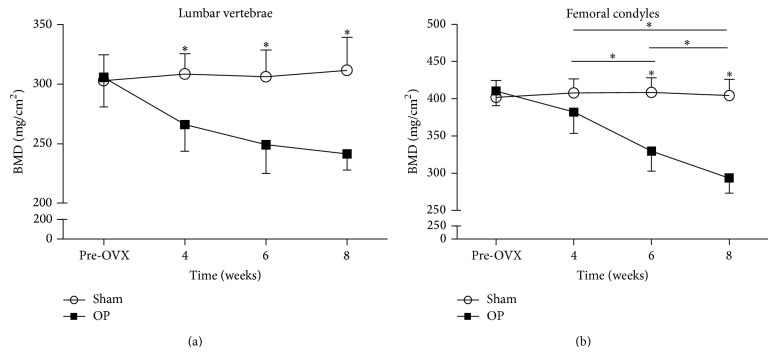
BMD values of the lumbar vertebrae and femoral condyles. (a) BMD values of the vertebrae. (b) BMD values of the femoral condyles. Asterisks showed the statistically significant difference between OP group and sham group at the same time point. Significant reductions in BMD of (a) lumbar vertebrae at 4 weeks and (b) femoral condyles at 6 weeks were seen in the OP group. Transverse lines with asterisks above showed that BMD values of (b) femoral condyles in OP group were significantly different among 4 weeks, 6 weeks, and 8 weeks. ^∗^ *P* < 0.05.

The BMD (mg/cm^2^) values of the lumbar vertebrae and femoral condyles were shown in Figure 2. Compared to the sham group, a significant reduction in BMD of lumbar vertebrae was seen in the OP group 4 weeks, 6 weeks, and 8 weeks after injection. The BMD of OP group at 8 weeks was reduced by 22.4% compared with sham group in vertebrae.

Compared to the sham group, a significant reduction in BMD of femoral condyles was seen in the OP group 6 weeks and 8 weeks after injection. The BMD of OP group at 8 weeks was reduced by 27.4% compared with sham group in femoral condyles.

The BMD values of the two anatomic sites were evaluated at different time points for the OP group. In vertebrae of OP group, BMD values among different time points were not statistically different. In the femoral condyles of OP group, BMD values among 4 weeks, 6 weeks, and 8 weeks were significantly different. However, the BMD values between pre-OVX and 4 weeks were not significantly different in femoral condyles. In addition, BMD of the sham group in the lumbar vertebrae and femoral condyles did not show any significant changes at any time point.

### 3.2. *μ*CT

**Figure 3:**
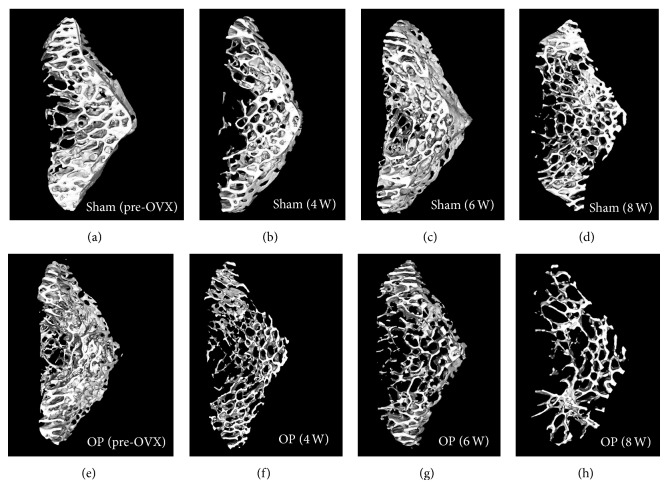
Three-dimensional trabecular microarchitectural images of rabbit vertebral bodies in two groups at different time points.

The 3D *μ*CT reconstructions of vertebral bodies showed lower bone volume in the trabecular bone and greater spacing in OP group compared with sham group (Figure 3). And as the experiment progressed, the difference became more obvious.

**Figure 4:**
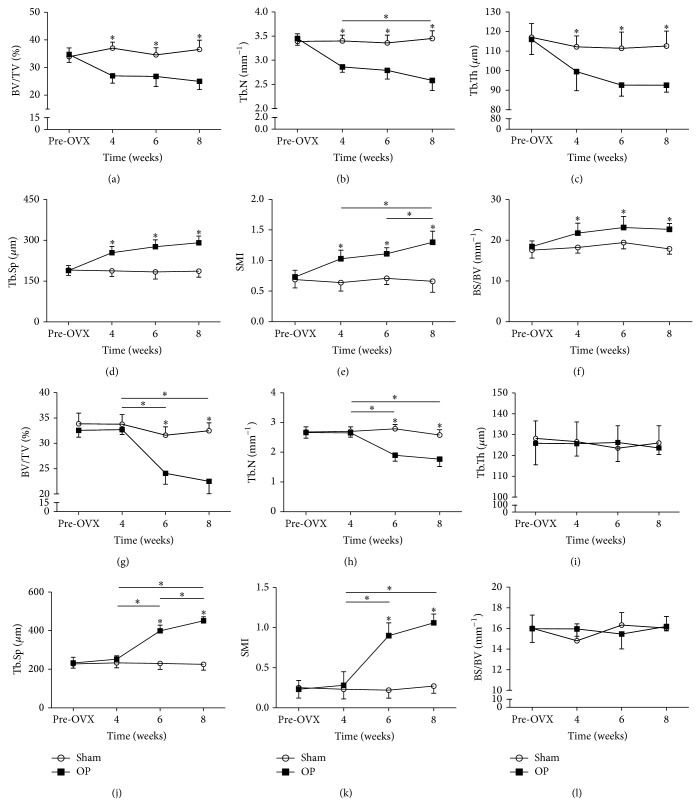
Overview of the microstructural parameters of L5 and femoral condyles. Structural parameters of vertebrae were (a) BV/TV, (b) Tb.N, (c) Tb.Th, (d) Tb.Sp, (e) SMI, and (f) BS/BV. Structural parameters of femoral condyles were (g) BV/TV, (h) Tb.N, (i) Tb.Th, (j) Tb.Sp, (k) SMI, and (l) BS/BV. Asterisks showed the statistically significant difference between OP group and sham group at the same time point. Transverse lines with asterisks above showed the differences in OP group among different time points. ^∗^ *P* < 0.05.

Changes in bone microarchitectural parameters of L5 were shown in Figure 4. All measured microarchitectural parameters of lumbar vertebrae in the OP group changed relative to the sham group at 4 weeks (BV/TV decreased by 27.1%, Tb.N decreased by 15.9%, Tb.Th decreased by 11.3%, Tb.Sp increased by 35.5%, SMI increased by 60.9%, and BS/BV increased by 19.3%), 6 weeks (BV/TV decreased by 22.5%, Tb.N decreased by 17.0%, Tb.Th decreased by 16.9%, Tb.Sp increased by 50.5%, SMI increased by 56.3%, and BS/BV increased by 19.1%), and 8 weeks (BV/TV decreased by 31.5%, Tb.N decreased by 25.2%, Tb.Th decreased by 17.8%, Tb.Sp increased by 56.1%, SMI increased by 97.0%, and BS/BV increased by 26.8%). Among the different time points in OP group at vertebrae, Tb.N in OP group of 4 weeks and 8 weeks was different. SMI of 6 weeks and 8 weeks, and 4 weeks and 8 weeks was different. No temporal changes in vertebral cancellous bone morphology were detected in the sham group.

Changes in bone microarchitectural parameters of femoral condyles were shown in Figure 4. Microarchitectural parameters, BV/TV, Tb.N, Tb.Sp, and SMI, of femoral condyles in the OP group changed relative to the sham group at 6 weeks (BV/TV decreased by 23.8%, Tb.N decreased by 31.9%, Tb.Sp increased by 73.4%, and SMI increased by 309.0%) and 8 weeks (BV/TV decreased by 30.7%, Tb.N decreased by 31.4%, Tb.Sp increased by 99.9%, and SMI increased by 292.6%). Among the different time points in OP group at femoral condyles, BV/TV of 4 weeks and 6 weeks, and 4 weeks and 8 weeks was different. Tb.N of 4 weeks and 6 weeks, and 4 weeks and 8 weeks was different. Tb.Sp of 4 weeks and 6 weeks, 6 weeks and 8 weeks, and 4 weeks and 8 weeks was different. SMI of 4 weeks and 6 weeks, and 4 weeks and 8 weeks was different. No temporal changes in femoral condylar cancellous bone morphology were detected in sham group.

### 3.3. Nanoindentation

**Figure 5:**
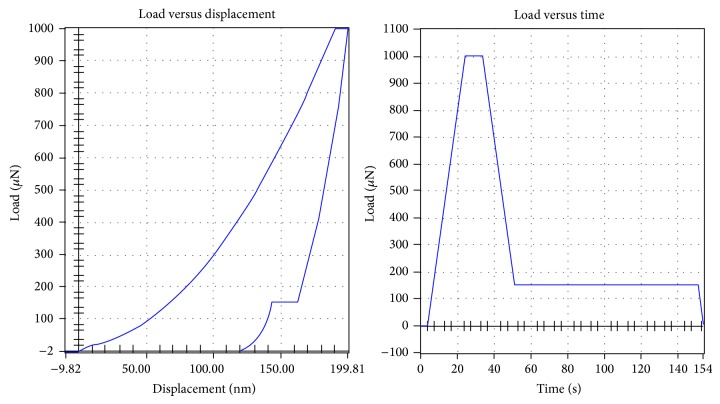
Load versus displacement and load versus time of nanoindentation test curves.

**Figure 6:**
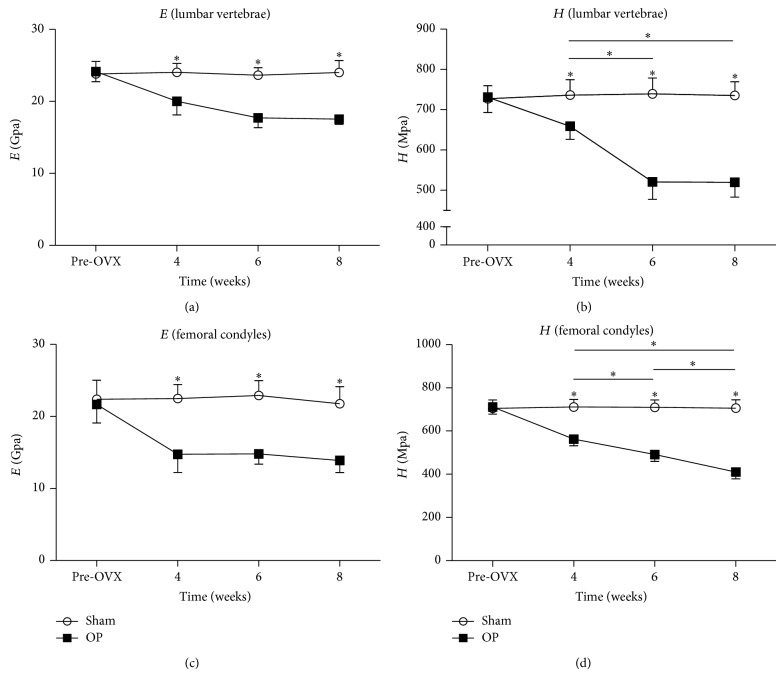
Nanoindentation results of ((a), (b)) vertebral and ((c), (d)) femoral condylar cancellous bone specimens. Asterisks showed the statistically significant difference between OP group and sham group at the same time point. Four weeks onwards the values of (a) elastic modulus and (b) hardness of lumbar vertebrae in the OP group decreased significantly. Similar results of (c) elastic modulus and (d) hardness also appeared in femoral condyles. Transverse lines with asterisks above showed the differences in OP group among different time points. ^∗^ *P* < 0.05.

Load versus displacement and load versus time of nanoindentation test curves were shown in Figure 5. Results of nanoindentation test were shown in Figure 6. Four weeks onwards the values of tissue elastic modulus and hardness of lumbar vertebrae in the OP group decreased significantly relative to the sham group. Among the different time points, significant differences were detected between 4 weeks and 6 weeks, and 4 weeks and 8 weeks of hardness in lumbar vertebral cancellous bone. No temporal changes in vertebral cancellous bone of tissue elastic modulus and hardness were detected in sham group.

Four weeks onwards the values of tissue elastic modulus and hardness of femoral condyles in the OP group decreased significantly relative to the sham group. Among the different time points, significant differences were detected between 4 weeks and 6 weeks, 6 weeks and 8 weeks, and 4 weeks and 8 weeks of hardness in femoral condylar cancellous bone. No temporal changes in femoral condylar cancellous bone of tissue elastic modulus and hardness were detected in sham group.

### 3.4. Mechanical Tests

**Figure 7:**
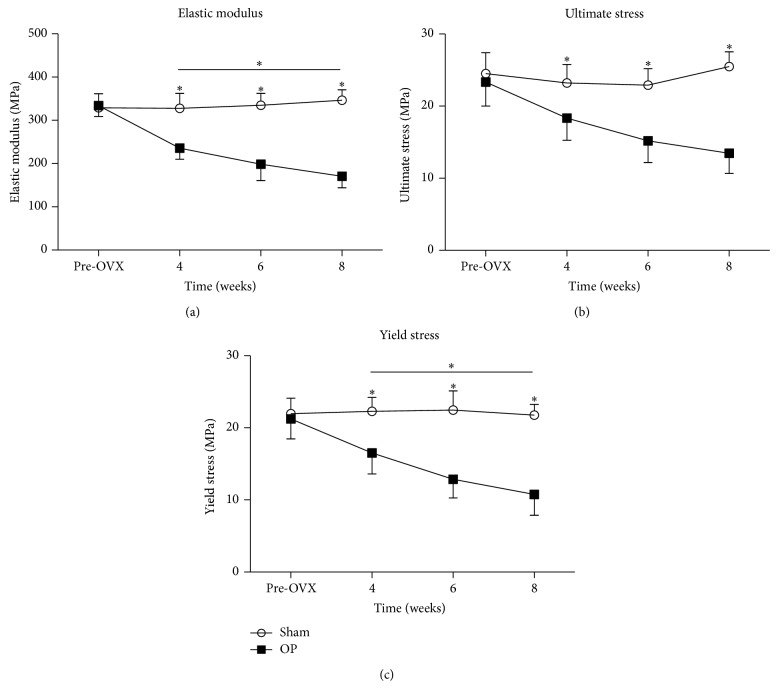
Biomechanical results of vertebral body specimens. Asterisks showed the statistically significant difference between OP group and sham group at the same time point. At 4, 6, and 8 weeks after injection in OP group, (a) the elastic modulus, (b) ultimate stress, and (c) yield stress were decreased significantly. Transverse lines with asterisks above showed the differences in OP group among different time points. ^∗^ *P* < 0.05.

The results of mechanical tests were shown in Figure 7. At 4, 6, and 8 weeks after injection in OP group, the elastic modulus was decreased by 28.1%, 40.7%, and 50.7%, the ultimate stress was decreased by 21.0%, 33.7%, and 47.2%, and the yield stress was decreased by 26.0%, 42.8%, and 50.6%, respectively, relative to sham group. Those values of the vertebrae in OP group exhibited a steady and progressive decreasing trend.

In OP group, the significant difference of elastic modulus existed between 4 weeks and 8 weeks, and the significant difference of yield stress existed between 4 weeks and 8 weeks. No temporal changes of elastic modulus, ultimate stress, and yield stress were detected in sham group.

## 4. Discussion

The main focus of this study was to examine temporal changes of cancellous bone quality to characterize an osteoporotic rabbit model and give more information about the influence of osteoporosis on nanomechanics. In this study, we used OVX combined with MPS administration to build a rabbit model of osteoporosis successfully and characterized cancellous bone features of this model as time went on. The full time length of the combined treatment was 8 weeks. During the period of combined treatment, the temporal changes of BMD, microarchitecture, nanomechanics, and biomechanics in the osteoporotic rabbits were assessed.

Compared with former OVX rabbits, this combined treatment could induce a more significant bone loss in vertebral cancellous bone in a shorter period. Jensen et al. [9] utilized OVX rabbits in their study. 27 weeks after OVX surgery, with regard to the vertebral cancellous bone, reduced bone volume, and trabecular numbers, significantly increased trabecular space was detected. However, trabecular thickness did not change, and BMD values of lumbar vertebrae were not examined in this study. In our study with regard to the microarchitecture of vertebral cancellous bone, all *μ*CT parameters (BV/TV, Tb.N, Tb.Th, Tb.Sp, SMI, and BS/BV) changed significantly at 4 weeks. It indicated that OVX + GC treatment could have more prominent effect on vertebral cancellous bone. In another study on OVX rabbits, there was a significant reduction of lumbar vertebra BMD (15.9%) in OVX rabbits compared to the sham group at 13 weeks after surgery [10]. In our study, the BMD of lumbar vertebrae in OP group decreased by 22.4% at the end of experiment. The decreasing proportion was larger, and the time length was shorter in our study.

In the current study, the temporal changes of cancellous bone quality were thoroughly studied. As time passed, the alteration of BMD, microarchitecture, and nanomechanical and biomechanical parameters were increasingly evident in OP group. The changes in these parameters manifested increasing severity in osteoporosis. This phenomenon indicated that the longer the OVX + GC (OVX combined with GC administration) treatment continued, the more severe the osteoporotic condition was. The same phenomenon was detected in rabbits treated with a combination of OVX with GC in former studies [12, 13].

With regard to the microarchitecture of the vertebral bodies and femoral condyles, the BV/TV could be thought as a 3D representation of BMD. And the changes of BV/TV values detected by *μ*CT were in accordance with the BMD values detected by DXA method in both anatomic sites. In vertebral bodies, all the Tb.N, Tb.Th, and Tb.Sp values changed significantly at 4 weeks, 6 weeks, and 8 weeks after injection. So the reduction of BMD values in vertebral bodies could be explained as the reduction of trabecular number and thickness. In femoral condyles, the Tb.N and Tb.Sp values changed significantly at 6 weeks and 8 weeks after injection. So the reduction of BMD values in femoral condyles could be explained as the reduction of trabecular number. This indicated that the deleterious alterations of microarchitecture in vertebral body and femoral condyles were different. Although these two anatomical sites were abundant in cancellous bone, greater and earlier changes were seen in vertebral bodies compared with femoral condyles. A similar result was also achieved in a sheep model of osteoporosis [22], and the greatest changes were seen in vertebral bodies.

The earliest time points, when we could detect significant changes of cancellous bone caused by osteoporosis in vertebral body and femoral condyles, were different. The BMD values of vertebral body in OP group decreased significantly at 4 weeks, while it decreased significantly at 6 weeks in femoral condyles. The earliest time points of this conventional method, DXA, to detect osteoporosis were different in these two anatomical sites. With regard to the microarchitecture of vertebral bodies, all values (BV/TV, Tb.N, Tb.Th, Tb.Sp, SMI, and BS/BV) of vertebral bodies changed significantly in OP group at 4 weeks, 6 weeks, and 8 weeks relative to sham group, whereas only BV/TV, Tb.N, Tb.Sp, and SMI values of femoral condyles in OP group changed significantly at 6 weeks and 8 weeks relative to sham group. The effects of OVX + GC on the BMD and microarchitecture of vertebral body and femoral condyles might be different. Lumbar vertebral cancellous bones seemed to be more sensitive to the combined treatment compared with femoral condyles in this rabbit model. It was consistent with humans also. Vertebral fractures could be the first clinical sign of osteoporosis [23] and to diagnose osteoporosis in its early stage; it is more sensitive to measure lumbar trabecular bone [24]. It would help diagnose osteoporosis at an early stage to select a proper anatomical site which can reflect the earliest osteoporotic changes [25].

Nanomechanical properties of cancellous bone detected by nanoindentation were modified in the osteoporotic rabbits from 4 weeks on. Our results were consistent with former studies in rats [26] and sheep [27]. In OVX rats, the tissue elastic modulus and hardness on nanomechanical level of cancellous bone in vertebrae were reduced significantly [26]. Tissue elastic modulus and hardness were reduced in cancellous bone in vertebral body in response to OVX as compared with sham (−11% for elastic modulus, −13% for hardness). And the decline in lumbar vertebral cancellous bone of OVX + GC rabbits in our study at 8 weeks for tissue elastic modulus was 26.9% and for hardness was 29.3%. The larger decreasing proportion in OVX + GC rabbits might be attributed to the fact that rabbits have faster bone turnover than other rodents [4–7]. Meanwhile, the magnitude of these changes in our study was the result of combined treatment of GC + OVX. In another study on OVX ovine model, tissue elastic modulus in the trabeculae of proximal femur was less than control ovine. But there were no statistically significant differences of hardness between OVX and control ovine. What calls for special attention is that the anatomical sites in this study were different from us, and the animal was also different [27]. The phenomena above in other studies could indicate that osteoporosis has effects on bone material level properties. Indeed, OVX increases both resorption and formation of bone; a combination of OVX and GC administration produces a significant increase in bone resorption and a significant decrease in bone formation. In addition to causing depletion of mature osteoblasts [28], GC alters the function of bone-forming cells by inhibiting the synthesis of type I collagen, which results in a decrease in bone matrix available for mineralization [29]. Those changes in nanoindentation results could be interpreted by a reduction in the mean degree of mineralization [30–32], a modification of crystal distribution and ultrastructure [33], and also bone collagen matrix alteration [34, 35], but without any change in mineral phase chemical composition [33, 35]. In contrast to our nanoindentation results, Guo and Goldstein [36] did not find any impact of OVX on tissue elastic modulus and hardness of bone in nanoindentation test. It might be that OVX alone could not have a detectable influence on tissue level mechanical properties of cancellous bone. Meanwhile, the tissue level mechanical properties of bone measured by nanoindentation depend not only on the sample itself, but also on the hydration state, probe geometry, and data analysis method, and it is complicated to compare values from different studies [37].

Nanoindentation was more sensitive to detect the changes of cancellous bone in this study than conventional methods (BMD and *μ*CT). There was no difference in BMD values and microarchitecture in femoral condyles between OP and sham group at 4 weeks, but tissue elastic modulus and hardness detected by nanoindentation were reduced significantly in OP group from 4 weeks to the end of experiment. Until 6 weeks after injection, significant changes of BMD and microarchitecture in femoral condylar cancellous bone emerged. And these differences were more obvious at 8 weeks. The main finding from this data was that a significant change of nanomechanical properties could be detected by nanoindentation before there were obvious changes of BMD and microarchitecture. Changes of BMD and microarchitecture lagged behind the nanomechanics of cancellous bone, and nanoindentation could help us detect the early changes of bone as osteoporosis develops.

Osteoporosis could reduce the mechanical resistance of bone which led to an increased risk of fracture. Axial compression test of lumbar vertebrae is recommended for testing the mechanical properties of cancellous bone [21]. Our normalized biomechanical parameters, including ultimate stress and yield stress, could assess the bone strength of vertebral bodies better than former studies [13, 14]. The mechanical data were normalized to obtain intrinsic biomechanical properties such as elastic modulus, ultimate stress, and yield stress, all of which are independent of size and shape [38]. In the present study, the deleterious effect of the combined treatment on bone strength was increased from 4 weeks on. At 8 weeks, bone strength was impaired worst. The significant change of microarchitecture detected by *μ*CT and nanomechanical properties detected by nanoindentation made contributions to the reduced bone strength. These results suggest that bone fragility is increased and that the bone is more susceptible to fracture in the OP group.

The limitation of our study was that we did not explore the single treatment, OVX or GC; how did they affect the BMD, microarchitecture, nanomechanics, and biomechanics of cancellous bone as time went on? More animal groups, labors, and material resources were needed to explore the single treatment in further studies. And the observation time was not long enough. In GC induced osteoporosis sheep, bone quality recovered with a rebound increase after cessation of GC [39]. However, in a sheep model of osteoporosis treated by a combination of OVX and GC, the osteopenia condition was not recovered even 6 months after cessation of GC [40]. Whether a prolonged GC treatment was needed to keep osteopenic bone in a rabbit model of osteoporosis was not decided in our study. Also, we did not explore the detailed mechanisms of how nanomechanics of cancellous bone were affected by the combined treatment.

In conclusion, our data demonstrated that temporal changes of microarchitectural and mechanical parameters of cancellous bone in the osteoporotic rabbit were significant. The temporal changes of cancellous bone in different anatomical sites may be different in the osteoporotic rabbits. As osteoporosis develops, the nanoindentation method could detect the change of bone quality at an earlier stage both at femoral condyle and at vertebra in the osteoporotic rabbit model than other conventional methods (*μ*CT, BMD).
